# Toward a universal decoder of linguistic meaning from brain activation

**DOI:** 10.1038/s41467-018-03068-4

**Published:** 2018-03-06

**Authors:** Francisco Pereira, Bin Lou, Brianna Pritchett, Samuel Ritter, Samuel J. Gershman, Nancy Kanwisher, Matthew Botvinick, Evelina Fedorenko

**Affiliations:** 1 Medical Imaging Technologies, Siemens Healthineers, Princeton, NJ 08540 USA; 2 Department of Brain and Cognitive Sciences, MIT, Cambridge, MA 02139 USA; 3 DeepMind, London, N1C 4AG UK; 4 Department of Psychology and Center for Brain Science, Harvard University, Cambridge, MA 02138 USA; 5 McGovern Institute for Brain Research, MIT, Cambridge, MA 02139 USA; 6 Gatsby Computational Neuroscience Unit, University College London, London, WC1E 6BT UK; 7 Department of Psychiatry, Harvard Medical School, Boston, MA 02115 USA; 8 Department of Psychiatry, Massachusetts General Hospital, Boston, MA 02114 USA

## Abstract

Prior work decoding linguistic meaning from imaging data has been largely limited to concrete nouns, using similar stimuli for training and testing, from a relatively small number of semantic categories. Here we present a new approach for building a brain decoding system in which words and sentences are represented as vectors in a semantic space constructed from massive text corpora. By efficiently sampling this space to select training stimuli shown to subjects, we maximize the ability to generalize to new meanings from limited imaging data. To validate this approach, we train the system on imaging data of individual concepts, and show it can decode semantic vector representations from imaging data of sentences about a wide variety of both concrete and abstract topics from two separate datasets. These decoded representations are sufficiently detailed to distinguish even semantically similar sentences, and to capture the similarity structure of meaning relationships between sentences.

## Abstract (web summary)

Previous work decoding linguistic meaning from imaging data has generally been limited to a small number of semantic categories. Here, authors show that a decoder trained on neuroimaging data of single concepts sampling the semantic space can robustly decode meanings of semantically diverse new sentences with topics not encountered during training.

## Introduction

Humans have the unique capacity to translate thoughts into words, and to infer others’ thoughts from their utterances. This ability is based on mental representations of meaning that can be mapped to language, but to which we have no direct access. The approach to meaning representation that currently dominates the field of natural language processing relies on distributional semantic models, which rest on the simple yet powerful idea that words similar in meaning occur in similar linguistic contexts^1^. A word is represented as a *semantic vector* in a high-dimensional space, where similarity between two word vectors reflects similarity of the contexts in which those words appear in the language^2^. These representations of linguistic meaning capture human judgments in diverse tasks, from meaning similarity judgments to concept categorization^3,4^. More recently, these models have been extended beyond single words to express meanings of phrases and sentences^5–7^, and the resulting representations predict human similarity judgments for phrase- and sentence-level paraphrases^8,9^.

To test whether these distributed representations of meaning are neurally plausible, a number of studies have attempted to learn a mapping between particular semantic dimensions and patterns of brain activation (see ref. ^10^ for a perspective on these techniques). If such a mapping can make predictions about neural responses to new stimuli, it would suggest that the underlying model has successfully captured some aspects of our representation of meaning. Early studies demonstrated the feasibility of decoding the identity of picture/video stimuli from the corresponding brain activation patterns, primarily in the ventral visual cortex^11–15^. More recently, studies have shown that similar decoding is possible for verbal stimuli, like words^16–22^, text fragments^23,24^, or sentences^25,26^. To represent meaning, these studies have used semantic features that were postulated by researchers, elicited from human participants, or inferred from text corpora based on patterns of lexical co-occurrence. The main limitation of these prior studies is the use of relatively small and/or constrained sets of stimuli, which thus leaves open the question of whether the models would generalize to meanings beyond those that they were built to accommodate. Further, the use of semantic features elicited from human participants (e.g., asking “is X capable of motion?”) is limited to concrete nouns (since we do not have models of what features characterize abstract nouns or verbs), and does not easily scale to a typical vocabulary of tens of thousands of words, or more than a small set of semantic features.

Here, we introduce an approach for building a universal brain decoder that can infer the meanings of words, phrases, or sentences from patterns of brain activation after being trained on a limited amount of imaging data. Our goal was to develop a system that would work on imaging data collected while a subject reads naturalistic linguistic stimuli on potentially any topic, including abstract ideas. Given the imaging data, the system should produce a quantitative representation of mental content—a semantic vector—that can be used in classification tasks or other types of output generation (e.g., word clouds).

Our decoder was trained on brain activation patterns in each participant elicited when they read individual words, and corresponding semantic vectors^27^. Our core assumption was that variation in each dimension of the semantic space would correspond to variation in the patterns of activation, and the decoder could exploit this correspondence to learn the relationship between the two. This was motivated by previous studies that showed that the patterns of activation for semantically related stimuli were more similar to each other than for unrelated stimuli^16,19^.The decoder then used this relationship to infer the degree to which each dimension was present in new activation patterns collected from the same participant, and to output semantic vectors representing their contents. If this relationship can indeed be learned, and if our training set covers all the dimensions of the semantic space, then any meaning that can be represented by a semantic vector can, in principle, be decoded.

The key challenge is the coverage of the semantic space by the words in the training set. This set is limited to a few hundred stimuli at most per imaging session as (i) multiple repetitions per word are needed because the functional magnetic resonance imaging (fMRI) data are noisy, and (ii) the stimuli need to be sufficiently separated in time given that the fMRI signal is temporally smeared. Ideally, we would obtain brain activation data for all the words in a basic vocabulary (~30,000 words^28^) and use them to train the decoder. Given the scanning time required, however, this approach is not practical. To circumvent this limitation, we developed a novel procedure for selecting representative words that cover the semantic space.

We carried out three fMRI experiments. Experiment 1 used individual concepts as stimuli, with two goals. The first was to validate our approach to sampling the semantic space by testing whether a decoder trained on imaging data for individual concepts would generalize to new concepts. The second goal was to comparatively evaluate three experimental approaches to highlighting the relevant meaning of a given word, necessary because most words are ambiguous. Experiments 2 and 3 used text passages as stimuli. Their goal was to test whether a decoder trained on individual concept imaging data would decode semantic vectors from sentence imaging data. The stimuli for both experiments were developed independently of those in experiment 1. In particular, for experiment 2, we used materials developed for a prior unpublished study, with topics selected to span a wide range of semantic categories. For experiment 3, we used materials developed by our funding agency, also designed to span diverse topics. Experiment 3 was carried out after our decoder was delivered to the funding agency, so as to provide an unbiased assessment of decoding performance.

We show that a decoder trained on a limited set of individual word meanings can robustly decode meanings of sentences, represented as a simple average of the meanings of the content words. These representations are sufficiently fine-grained to distinguish even semantically similar sentences, and capture the similarity structure of the inter-sentence semantic relationships.

## Results

### Parcellation and sampling of the semantic space

**Fig. 1:**
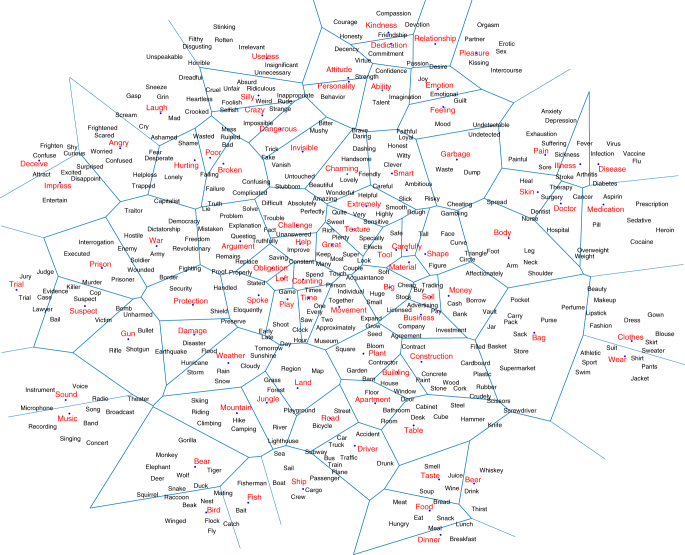
Visualization of semantic space. Two-dimensional visualization of 80 regions of 300-dimensional semantic space, containing points for 80 of the 180 target words used in experiment 1 (in red), surrounded by 5 words from the same cluster (in black). The blue lines are an approximation of region boundaries as determined by the clustering algorithm

We obtained semantic vectors for all words in a basic vocabulary of approximately 30,000 words, selected from ref.^28^. We used 300-dimensional GloVe vectors, as this representation proved to be superior to others in experiments predicting behavioral data^4^. We then used spectral clustering^29^ to group words into 200 regions (semantic clusters, see subsection “Spectral clustering of semantic vectors” in Methods). Almost all regions (clusters) that resulted from this procedure were intuitively interpretable. Some corresponded to classic concrete concept categories (e.g., dwellings or body parts); others were more abstract (e.g., virtues or states of mind/emotions); yet others did not fit into any of the classic categories, but lent themselves easily to characterization (e.g., size/numerosity or manners of speaking). We excluded 20 regions for lack of interpretability; these contained either highly infrequent words (resulting in poor semantic vector estimates) or extremely common ones (resulting in uninformative semantic vectors). We then hand-selected representative words from each of the remaining 180 regions, which were used in creating the stimuli for experiment 1 (see subsection “Design of fMRI experiment 1 on words” in Methods). A subset of regions and selected words are shown in Fig. 1.

### Experiment 1 on single concept decoding

**Fig. 2:**
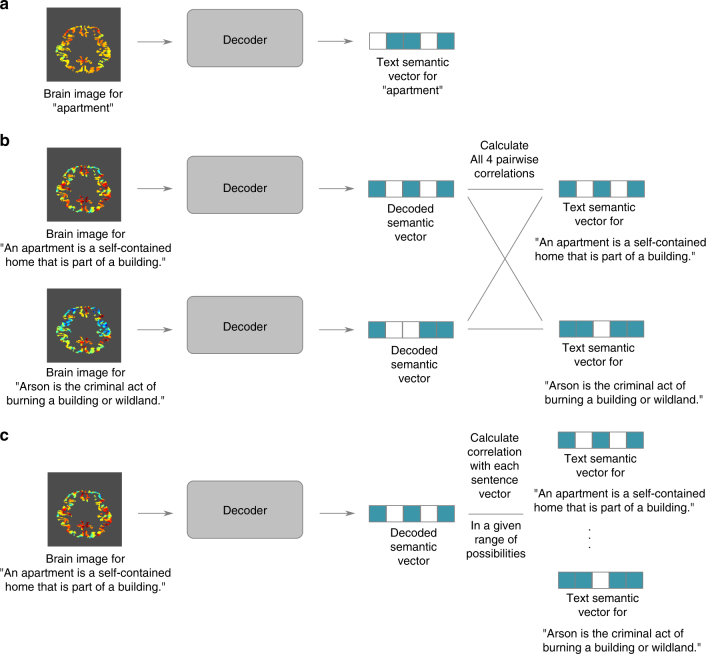
Decoder schematic. **a** The decoder is trained to take a brain image as input and output the corresponding text semantic vector, for many different image/vector pairs. **b**,** c** The decoder is applied to new brain images and outputs decoded semantic vectors, which are then evaluated against text semantic vectors. **b** A pairwise evaluation, which is correct if vectors decoded from two images are more similar to the text semantic vectors for their respective stimuli than to the alternative. **c** A rank evaluation, where the decoded vector is compared to the text semantic vectors for a range of stimuli

Our decoder is trained on brain activation patterns in each participant elicited by individual words and corresponding semantic vectors (Fig. 2a). The decoder uses the learned relationship to infer the degree to which each dimension is present in new activation patterns collected from the same participant, and outputs semantic vectors representing their contents (Fig. 2b). Each dimension is predicted using a different ridge regression, with parameters estimated from training data (see “Decoding methodology” in Methods).

**Fig. 3:**
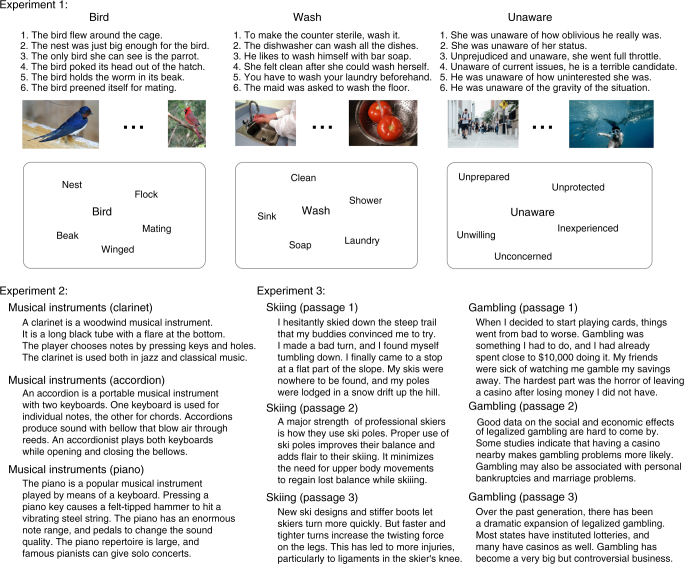
Examples of stimuli from experiments 1, 2 and 3. **a** Examples of stimuli used for sample words (a noun, a verb, and an adjective) in experiment 1. Each word was presented in three paradigms (in a sentence, with an image, or with five related words), with multiple repetitions in each paradigm. Cardinal image courtesy of Stephen Wolfe. Barn swallow image courtesy of Malene Thyssen. **b** Examples of stimuli used in experiments 2 and 3. In experiment 2, each passage belongs to a subtopic (e.g., “clarinet”) of one of the 24 main topics (e.g. “musical instruments”); in experiment 3, there are three different passages for each one of the 24 main topics. Note that sentences are presented on the screen one at a time

The brain imaging data used to build the decoder were collected in experiment 1 and focused on single concepts. We use the term concepts instead of words because words were presented so as to target a particular meaning, given that most words are ambiguous. We scanned 16 participants in three paradigms (Fig. 3), all aimed at highlighting the relevant meaning of each of 180 words (128 nouns, 22 verbs, 29 adjectives and adverbs, and 1 function word), selected (as described in “Stimulus selection and semantic space coverage” in Methods). In the first paradigm, the target word was presented in the context of a sentence that made the relevant meaning salient. In the second, the target word was presented with a picture that depicted some aspect(s) of the relevant meaning. In the third, the target word was presented in a “cloud”, surrounded by five representative words from the cluster. These paradigms were chosen over a simpler paradigm where the target word appears in isolation because words are highly ambiguous, especially outside the realm of concrete nouns. These paradigms ensure that the subject is thinking about the relevant (intended) meaning of each word. For each concept, we combined stimulus repetitions (4–6 for each paradigm) using a general linear model to produce one brain image per paradigm per participant (restricted to a gray matter mask of ~50,000 voxels^30^). For some analyses, including the decoding analyses for experiments 2 and 3, we further averaged the images for each concept across the three paradigms (see subsection “fMRI data acquisition and processing” in Methods).

We evaluated the decoder by training it and testing it using different subsets of the 180 concepts in a cross-validated manner. For each participant, we iteratively partitioned data into 170 concepts for training a decoder, and 10 left-out concepts for testing it. In each such partition, we selected 5000 voxels (~10% of the total) by the degree to which they could predict semantic vectors for the 170 training concepts; we then trained the decoder using those voxels (see “Decoding methodology” in Methods). For each left-out concept, a semantic vector was decoded from the corresponding activation pattern, resulting in 180 decoded vectors, after all iterations had taken place. We carried out this procedure using the data from each paradigm separately or the average of the three paradigms.

Decoding performance was evaluated in two ways. The first was a pairwise classification task where, for each possible pair of words, we computed the similarity between the decoded vectors and the “true” (text-derived) semantic vectors (Fig. 2b, right). If the decoded vectors were more similar to their respective text-derived semantic vectors than to the alternative, we deemed the classification correct. The final accuracy value for each participant is the fraction of correct pairs. The second was a rank accuracy classification task where we compared each decoded vector to all 180 text-derived semantic vectors, and ranked them by their similarity (Fig. 2c, right). The classification performance reflects the rank of the text-derived vector for the correct word: 1 if it is at the top of the rank, 0 if it is at the bottom, and in-between otherwise. The final accuracy value for each participant is the average rank accuracy across the 180 concepts. The null hypothesis value (chance performance) is 0.5 for both measures, but the statistical tests are different (see subsection “Statistical testing of results” in Methods).

**Fig. 4:**
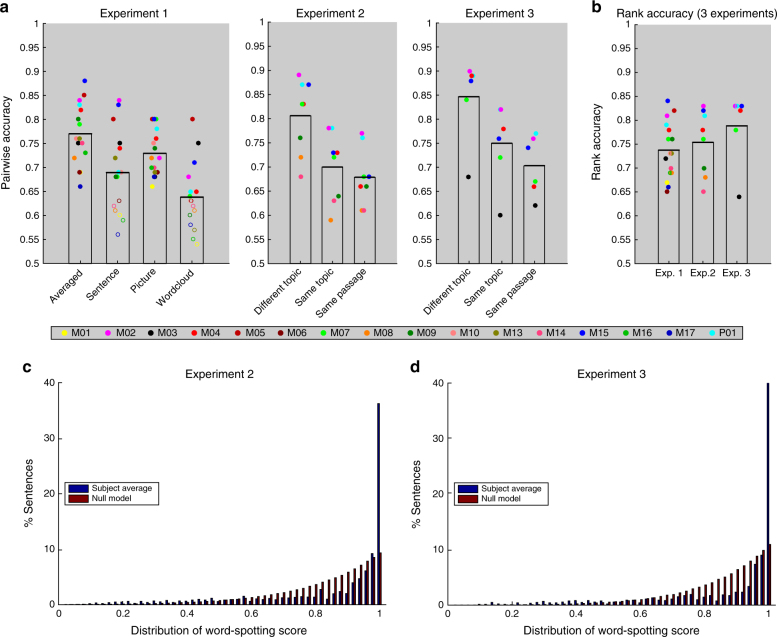
Classification results. **a** Pairwise accuracy results in experiments 1 (*n* = 16, left), 2 (*n* = 8, middle), and 3 (*n* = 6, right). For experiment 1, we report the results for each of the three paradigms separately, as well as averaged across paradigms. For experiments 2 and 3, we report three measures: classifying sentences from (i) different topics (left), (ii) different passages within the same topic (middle), and (iii) the same passage (right). Each dot represents pairwise accuracy for an individual subject. Dots of the same color refer to the same individual across experiments. Filled dots (cf. empty dots) represent significant results. **b** Rank accuracy results in experiments 1 (180 word choices), 2 (384 sentence choices), and 3 (243 sentence choices). **c** The distribution of word-spotting results in experiment 2, on average across subjects, contrasted with the distribution under a simulated null model. **d** The same for experiment 3

We robustly classified left-out concepts for each of 16 participants when using the images averaged across the three paradigms or when using the picture paradigm, for 10 of the 16 participants when using the sentence paradigm, and for 7 of the 16 participants when using the word cloud paradigm (mean accuracies: 0.77, 0.73, 0.69, and 0.64; all significant results have *p*-values < 0.01, using a conservative binomial test with Bonferroni correction for the number of participants (16) and experiments (4); Fig. 4a). The average rank accuracy (when using the images averaged across the three paradigms) was 0.74 (all significant results have *p*-values < 0.01 using a test based on a normal approximation to the null distribution, with Bonferroni correction for the number of participants (16) and experiments (3); results are shown in Fig. 4b, *p*-values for each subject/task are provided in Supplementary Table 1).

### Experiments 2 and 3 on sentence decoding

**Fig. 5:**
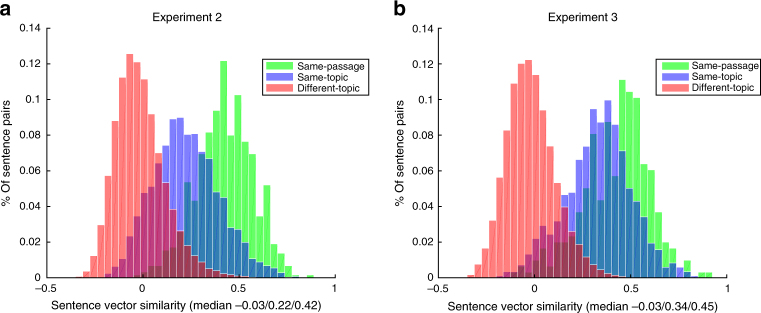
Distribution of correlation values between semantic vectors for stimulus sentences in experiments 2 and 3. **a** The distribution of correlation values between the text-derived semantic vectors for pairs of sentences from different topics (red), from different passages within the same topic (blue), and from the same passage (green) for experiments 2. **b** Same for experiment 3

Given that experiment 1 demonstrated that our approach could generalize to novel concepts, we carried out two further experiments to test the decoding of sentence meanings using stimuli constructed independently of the materials in experiment 1 and of each other. In experiment 2, we used a set of 96 text passages, each consisting of 4 sentences about a particular concept, spanning a broad range of content areas from 24 broad topics (e.g., professions, clothing, birds, musical instruments, natural disasters, crimes, etc.), with 4 passages per topic (e.g., clarinet, accordion, piano, and violin for musical instruments; Fig. 3). All passages were Wikipedia-style texts that provided basic information about the relevant concept. In experiment 3, we used a set of 72 passages, each consisting of 3 or 4 sentences about a particular concept. As in experiment 2, the passages spanned a broad range of content areas from 24 broad topics, unrelated to the topics in experiment 2 (e.g., skiing, dreams, opera, bone fractures, etc.), with 3 passages per topic (Fig. 3). The materials included Wikipedia-style passages (*n* = 48) and first-/third-person narratives (*n* = 24). The two experiments were comparable in their within- and between-passage/topic semantic similarities (Fig. 5). Each passage—presented one sentence at a time—was seen 3 times by each participant, in both experiments 2 and 3. Each sentence was presented for 4 s, with 4 s between sentences, which allowed us to obtain brain images for individual sentences.

A decoder (identical to that used in experiment 1, trained on all available data) was trained—for each participant separately—on the brain images for the 180 concepts from experiment 1 (using the average of the three paradigms), with 5000 most informative voxels selected as described above. The decoder was then applied to the brain images for the sentences from experiments 2 and 3, yielding a semantic vector for each sentence. A text-derived semantic vector for each sentence was created by averaging the respective content word vectors^5^.

The decoded vectors were evaluated in three progressively harder pairwise classification tasks, with sentences coming from (i) different topics (e.g., a sentence about an accordion vs. a butterfly), (ii) different passages within the same topic (e.g., a sentence about an accordion vs. a clarinet), and (iii) different sentences from the same passage (e.g., two sentences about an accordion), for all possible pairs in each task. As shown in Fig. 4a, we could distinguish sentences at all levels of granularity in experiment 2 (all 8 participants have *p*-values < 0.01 after Bonferroni correction on task i, task ii, and task iii, *p*-values for each subject/task are provided in Supplementary Table 2), and experiment 3 (all 6 participants have *p*-values < 0.01 after Bonferroni correction on for task i, task ii, and task iii, *p*-values for each subject/task are provided in Supplementary Table 3), using the same conservative testing approach as in experiment 1.

To ensure that the pairwise accuracy results were not due to differences in the number of content words across sentences, we carried out a control analysis. For each pair of sentences, we calculated the fraction of times it was classified correctly across subjects. We then grouped sentence pairs by the difference in the number of content words (range: 0–9 words), and calculated the average pairwise accuracy for each set. The average pairwise accuracy was roughly the same across sets (0.81 and 0.84, on average, in experiments 2 and 3, respectively; Supplementary Figure 1); only for the pairs with the most extreme differences (5 or more words), which constitute a minuscule fraction of the total pairs (1–2%), do we see a slight increase (average of 0.84/0.85).

We also calculated the rank accuracy over 384 and 243 sentences in experiments 2 and 3, respectively, which were significant for all subjects (mean accuracy: 0.76 and 0.79; all significant *p*-values < 0.01, using a test based on a normal approximation to the null distribution, with Bonferroni correction for the number of participants and experiments; results are shown in Fig. 4b, *p*-values for each subject/task are provided in Supplementary Tables 2 and 3). Rank accuracy is particularly informative here given the large number of closely related sentences within passages and topics. This is because it assigns full credit to getting the exact stimulus sentence at the top of the ranking, and partial credit if the top-ranked sentence is semantically related to the target.

Finally, we performed an open-ended decoding task to determine how well the decoded vectors can be used to identify the content words in the stimulus sentences. This analysis quantifies the degree to which the decoder retrieves relevant information when we do not have a closed set of ground-truth stimuli to choose from. In this task, the words in a basic vocabulary (*n* = ~30,000) were ranked according to how similar their vectors are to the decoded vector. For each sentence, we calculated a word-spotting score, defined as the best rank accuracy of any of the words in the sentence. This is a challenging task for two reasons. First, the decoded vector represents mental contents as an entire sentence is processed, often in the context of preceding sentences, rather than individual words. Hence, the decoder will succeed only if the sentence activation pattern resembles those of individual words. Second, this ranking considers synonyms of the words in the sentence, as well as related words, names of the categories the words belong to, etc. All of these compete with and may be ranked higher than the actual word in the sentence. For each subject, we computed the distribution of scores across all sentences in experiments 2 and 3, and compared it with a simulated null distribution (for every sentence, take the maximum of randomly generated ranks). Figure 4c, d plots the average distribution across subjects against this null distribution for experiments 2 and 3, respectively. The distributions were significantly different from the null distribution for 7 of 8 participants in experiment 2 and all 7 participants in experiment 3. The median rank accuracy of the best ranked word is 0.96 in both experiments (all significant *p*-values < 0.01 using a 2-sided Kolmogorov–Smirnov test, with Bonferroni correction for the number of participants). The median scores in the null model are 0.86 and 0.88, but with a very different distribution. The highest ranked word is in the top 1% for 24% of the sentences in experiment 2 and 33% of the sentences in experiment 3 (median across subjects).

**Fig. 6:**
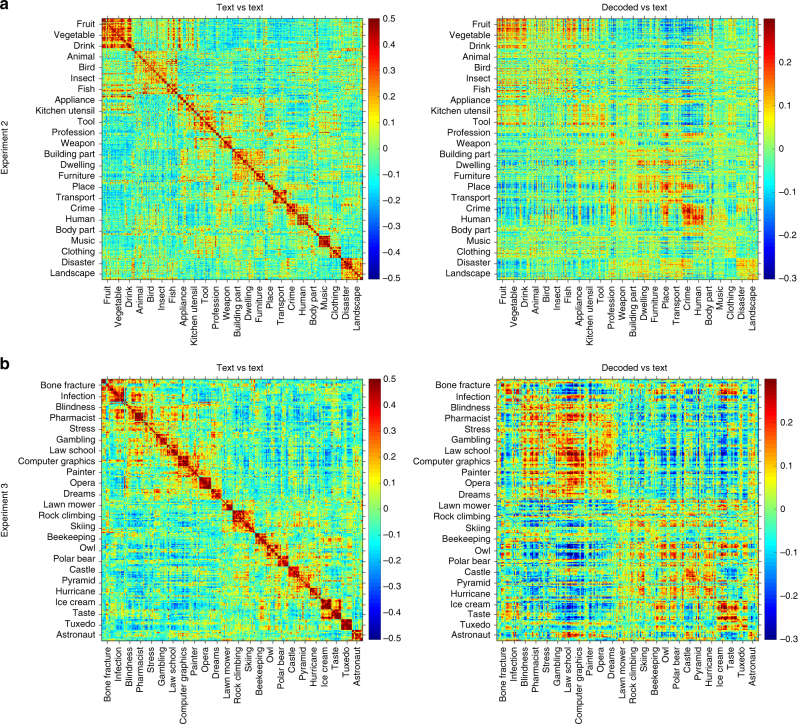
Similarity structure between text-derived and decoded semantic vectors. **a** Experiment 2 (384 sentences): correlations between the text-derived vectors (left) and between text-derived and decoded vectors, averaged across subjects (right). The sentences are sorted so that sentences in the same passage are adjacent, passages in the same topic are adjacent, and related topics are adjacent (only topic labels are shown for readability). **b** Corresponding plots for experiment 3 (243 sentences)

In addition to the evaluation tasks, we tested whether the similarity structure of the decoded semantic space mirrors the similarity structure of the text-derived one. In particular, in both experiments 2 and 3, sentences within a passage have some similarity to each other given that they all talk about the same concept. Further, sentences in passages within the same topic bear some similarity to each other. Finally, some of the topics are related (e.g., “dwellings” and “building parts”, or “bone fracture” and “infection”). Figure 6 shows the similarity (correlation) matrix between decoded and text vectors for all sentences, averaged across subjects, and contrasts it with the similarity matrix between all text vectors (histograms of values on the diagonal are shown in Supplementary Figure 2). We have previously found that the correlation between text-derived semantic vectors predicts behavioral relatedness^4^. Here, we found that it also predicts the relatedness among the vectors decoded from fMRI data, as evidenced by reliable correlations between corresponding entries in the text-derived and decoded matrices (Fig. 6; the average correlation between decoded/text and text/text similarity matrix entries across participants is *r* = 0.32 in experiment 2 and *r* = 0.39 in experiment 3; both are significantly different from 0, *p*-value < 0.01).

Finally, we replicated the pairwise and rank accuracy analyses using skip-thought vectors^7^ instead of GloVe vectors. This representation produces vectors for sentences directly, rather than as an average of vectors of content words (see subsection “Semantic vectors” in Methods for details). The results were virtually the same as those obtained with GloVe, and are shown in Supplementary Figure 3.

### Spatial distribution of informative voxels

**Fig. 7:**
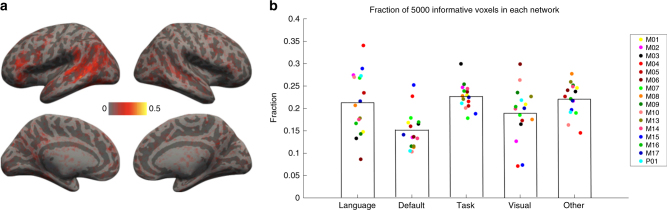
Distribution of informative voxels across the brain. **a** The fraction of subjects, out of the 16 subjects used in experiment 1, where each voxel was among the 5000 informative voxels that were used to train a decoder. **b** The fraction of the 5000 informative voxels belonging to each of the four networks described in the text, as well as the rest of the brain

The decoder uses the 5000 most informative voxels in each subject, chosen without any location constraint (aside from gray matter masking). The location of these voxels was consistent across participants, as shown in Fig. 7a, where the value of each voxel is the fraction of 16 participants for whom that voxel was among the 5000 most informative. Approximately 10,000 unique voxels appear in at least one participant, with approximately 5000 appearing in 4 or more. Some variability in the locations of informative voxels is to be expected (e.g., see refs.^31–33^).

Given the consistency in the location of informative voxels across participants, we asked how these voxels are distributed among the large-scale brain networks that have been linked to high-level cognition and/or semantic processing specifically: (i) the frontotemporal language-selective network^34^, (ii) the default mode network (DMN)^35,36^, (iii) the task-positive (TP) network^31,36,37^, as well as (iv) the visual network from ref.^31,35^. The language network was defined by the contrast between the reading of sentences and nonword lists based on 220 activation maps, bilateralized^34^, and the other three networks were defined based on resting-state functional correlation data from ref. ^31^. The language network has little to no overlap with the other three networks (overlap=number of voxels in common/number of voxels in either, 0.11 (DMN), 0.05 (TP), and 0.02 (visual), respectively). A priori, the language network and the DMN may seem like the most likely candidates for containing informative voxels, given that the language network plausibly stores the mappings between linguistic forms and meanings (which are used to both interpret and generate linguistic utterances), and the DMN has been linked to semantic processing based on meta-analyses of fMRI studies^36^. However, as Fig. 7b shows, the 5000 informative voxels are roughly evenly distributed among the four networks and other brain locations (language 21%, DMN 15%, TP 23%, visual 19%, other locations 22%, on average across participants). Note, however, that the language network is substantially smaller than the others (Table 1, 4670 voxels in the language network versus 6490, 11,630, and 8170 for the other three networks, and approximately 20,000 for the rest of the brain, on average across participants), and thus contains a relatively higher proportion of informative voxels.

**Table 1:** Average decoding performance across subjects when restricting voxel selection to different networks

		Brain	Language	Default	Task	Visual	Other
	Approximate no. of voxels	~50K	4670	6490	11,630	8170	~20K
	(average across 16 subjects)			(Power 2011, minus any language overlap)			
Experiment 2	Pairwise: different topic	0.81	0.75	0.69	0.74	0.65	0.73
	Pairwise: different passage	0.70	0.67	0.60	0.65	0.58	0.63
	Pairwise: same passage	0.68	0.64	0.60	0.63	0.55	0.62
	Rank: 384 sentences	0.76	0.70	0.65	0.69	0.62	0.67
Experiment 3	Pairwise: different topic	0.85	0.80	0.73	0.77	0.69	0.78
	Pairwise: different passage	0.75	0.72	0.65	0.67	0.62	0.69
	Pairwise: same passage	0.70	0.67	0.60	0.64	0.58	0.64
	Rank: 243 sentences	0.79	0.74	0.68	0.72	0.65	0.72

Top row: Approximate numbers of voxels for the whole brain, language network, the three Power 2011 networks described in the text, and the rest of the brain after excluding those four networks. Bottom rows: Average decoding performance across subjects, after restricting the choice of 5000 voxels to each of the four networks, or the rest of the brain, using the four measures described in the text. For default, task, and visual, we excluded voxels belonging to the language network. Given its small size, (almost) all its voxels were used in all the subjects

Finally, we examined whether decoding can be performed using only information contained in each of the networks above. To do this, we restricted the selection of 5000 voxels to each network or other brain locations and re-ran the decoding analyses for experiments 2 and 3. As shown in Table 1, the average decoding performance across subjects in the three classification tasks was only slightly impaired if selecting from the language and task-positive networks, and much more so for DMN and visual networks. The *p*-values of one-tailed paired *t*-tests comparing performance of the whole-brain baseline against each selection are given in Supplementary Table 4; significant results had *p*-values < 0.01, with Bonferroni correction to take into account the number of tasks, networks being compared, and experiments (see Methods for further details). Overall, this suggests that the voxels that contribute to decoding are widely distributed. Further, the fact that the majority of informative voxels fall outside of the visual cortices suggests that the decoded information is not primarily visual in nature (i.e., does not result from low-level visual features of the stimuli or the imagery associated with sentence comprehension) and instead encodes semantic features.

## Discussion

Across three fMRI experiments, we show that training a decoder on single concepts that comprehensively cover the semantic space leads to the ability to robustly decode meanings of semantically diverse new sentences. As described above, several previous studies have demonstrated the ability to perform classification tasks on verbal stimuli^16–26^. In those studies, as well as ours, the model building (training) stage consists of learning a relationship between the representations of input stimuli and the imaging data. The decoding models are then used to either predict the imaging data (on a voxel-by-voxel basis) for test stimuli, as in the other studies, or to predict the representation of the stimulus shown when test imaging data were acquired, as we do here. Both of these approaches allow classification tasks to be performed, but only the latter produces a representation that is directly usable for other, more sophisticated, tasks (e.g., generating a word cloud output).

Our work goes substantially beyond prior work in three key ways. First, we develop a novel sampling procedure for selecting the training stimuli so as to cover the entire semantic space. This comprehensive sampling of possible meanings in training the decoder maximizes generalizability to potentially any new meaning. Indeed, we were able to decode diverse concepts from dozens of semantic categories, including abstract concepts (e.g., ignorance) and spanning objects/ideas (e.g., pleasure), actions (e.g., cook), psychological events/states (e.g., deceive), and object/action properties (e.g., deliberately). We used an ad-hoc approach for selecting a concept from each region of the semantic space, but one can easily envisage automated procedures for stimulus selection or generation (e.g., using text fragments containing the words from each region).

Second, we show that although our decoder is trained on a limited set of individual word meanings, it can robustly decode meanings of sentences represented as a simple average of the meanings of the content words. Further, this decoding is characterized by a relatively fine semantic resolution, producing distinct representations, even for sentences that deal with similar meanings (e.g., two sentences about an accordion). To our knowledge, this is the first demonstration of generalization from single-word meanings to meanings of sentences.

Third, we test our decoder on two independent imaging datasets, in line with current emphasis in the field on robust and replicable science^33^. The materials (constructed fully independently of each other and of the materials used in the training experiment) consist of sentences about a wide variety of topics—including abstract ones—that go well beyond those encountered in training.

Research on the neural basis of semantic representations is still in its relative infancy: there are no clearly articulated hypotheses about how concepts are represented and combined, which could be empirically tested against one another. The dominant proposals make localizationist claims that conceptual representations are focally represented in, e.g., the anterior temporal lobes (see, e.g., refs. ^31,38^) or in parts of the angular gyrus (see, e.g., ref. ^36^), or argue that semantic processing draws on sensory and motor cortices (see, e.g., ref. ^39^). Along with some earlier studies (see, e.g., refs. ^16,22–24^), our work shows that semantic information is distributed across several high-level cortical networks rather than being restricted to a particular brain region or to sensory and motor cortices.

Our work demonstrates the viability of using distributed semantic representations to probe meaning representations in the brain, laying a foundation for future development and evaluation of precise hypotheses about how concepts are represented and combined. For example, here we used word vectors, which represent multiple meanings (since most words are ambiguous). Vectors for specific meanings have recently become available^40–42^ and may provide more precise mapping between brain activation and semantic dimensions. This specificity will also make it simpler to automatically identify viable stimuli for use in imaging experiments for training decoders, obviating the need for manual intervention. Further, decoding quality varies across semantic dimensions. For this initial evaluation, we chose to minimize the number of free parameters and to favor simplicity of the approach. However, we expect that decoding performance will improve by selecting different sets of informative voxels for different dimensions, and possibly different regression models depending on the distribution of values in each dimension. This approach can shed light on how different dimensions are spatially organized in the brain. Finally, our approach can be used with *any* distributional semantic model, including future ones capable of expressing more subtle meaning distinctions (e.g., those that depend on word order and hierarchical relationships among words, see, e.g., refs. ^43,44^), or additional information such as frame semantics for a sentence^45^. Although we did not see improved performance for one version of such a model (skip-thought vectors^7^), this may reflect the fact that vectors derived from this model have a more complex structure than GloVe (see subsection “Semantic vectors” in Methods for details); future attempts with different models may prove more fruitful.

In summary, we report a viable approach for building a universal decoder, capable of extracting a representation of mental content from linguistic materials. Given the progress in the development of distributed semantic representations, we believe that the semantic resolution of brain-based decoding of mental content will continue to improve rapidly. Our hope is that our work will serve as one of the cornerstones for developing and testing specific proposals about the nature of concepts, the organizing principles of the semantic space, and the computations that underlie concept composition.

## Methods

### Semantic vectors

The notion of a semantic vector was initially introduced with Latent Semantic Analysis^2^ in 1997, but the most commonly used types were not available until recently. We first carried out a comparison of all types of semantic vectors available with regard to how well they could predict human judgments on behavioral tasks, published in ref. ^4^; see also ref. ^3^. Two of the semantic vector representations were superior to others: word2vec^46^ and GloVe^27^. Both methods generate 300-dimensional vectors to represent each word by exploiting the statistics of word co-occurrences in a certain context (typically the span of a sentence, or a window of 5–10 words), tabulated over corpora with tens of billions of words. In word2vec, the representation learned is such that the identity of a word can be predicted given the average semantic vector of the other words in the context (e.g., the 5 words before and the 5 words after). In GloVe, the representation learned is derived directly from a normalized version of the matrix of word co-occurrences in context. We opted for GloVe for practical reasons, like the homogeneity of value ranges in different dimensions and the vocabulary size, but decoding performance was similar with word2vec vectors. A number of other semantic representations have been put forward recently, but improvements in behavioral or decoding performance have been marginal at best.

Generating vectors to represent sentences is typically done by averaging vectors for the content words (after dereferencing pronouns)^5^, as we did in this paper. There is only one method for generating vectors for sentences directly that is in widespread use, skip-thought vectors. This method aims to represent a sentence in a vector containing enough information to reconstruct the ones preceding and succeeding it in the training text where it occurs. Skip-thought vectors differ from GloVe in their high-dimensionality—4800 dimensions rather than 300—and heterogeneity, since dimensions have very different distributions of values instead of being roughly Gaussian distributed. From an engineering standpoint, it is more complicated to decode them from imaging data, since ridge regression models are not appropriate for many of the dimensions. Vector comparison between decoded and text-derived vectors is also more involved, since some dimensions are much more influential than others in the computation of typical measures such as correlation or Euclidean distance. We did reproduce our analysis using skip-thought vectors, with results shown on Supplementary Figure 3; using the same decoder and procedures as with GloVe vectors, the results were virtually the same.

### Spectral clustering of semantic vectors

In order to select words to use as stimuli, we started with GloVe semantic vectors for the 29,805 basic vocabulary words in ref.^28^ for which vectors were available. We then carried out spectral clustering^29^ of those vectors to identify 200 regions (clusters) of related words. We used spectral clustering for two reasons. The first is that traditional clustering algorithms like *k*-means implicitly assume multivariate Gaussian distributions for each cluster with the same covariance structure by using Euclidean distance between vectors. It is unclear whether this assumption is reasonable here, and our early attempts that used *k*-means produced many clusters that were not readily interpretable. The second reason is that the experiments described in refs. ^3,4^ showed that cosine similarity (or correlation) between the semantic vectors reflects semantic relatedness. Spectral clustering uses the cosine similarity between words to place them in a new space where words with the same profile of (dis)similarity to *all* other words are close together, and then performs *k*-means in this new space. This is, intuitively, more robust than simply comparing the vectors of any two words when grouping them together, and the results subjectively bore it out (in terms of the interpretability of the resulting clusters).

The spectral clustering procedure consisted of these steps:

1. calculate a 29,805 by 29,805 matrix *C* with the cosine similarity for each pair of vectors;
2. normalize *C* from (1) to fall in a 0–1 range $\left( {C \leftarrow \frac{{C + 1}}{2}} \right)$, and zero out the diagonal;
3. normalize *C* from (2) so that each row adds up to 1;
4. compute a 100-dimensional eigendecomposition *D* of *C* from (3);
5. run *k*-means (*k* = 200) on *D* using squared Euclidean distance and the *k*-means ++ algorithm (*k*-means ++ is a more stable version of *k*-means, used by default in MATLAB).

### Stimulus selection and semantic space coverage

The number of clusters sought was determined by the maximum number of stimuli that could be fit into a single scanning session, with up to 6 repetitions (median cluster size 150 words, range 81–516). As discussed in the main text, almost all the 200 clusters were easy to interpret. A small percentage (~10%) were harder to interpret; these tended to contain (i) infrequent, unrelated words or (ii) extremely frequent, uninformative words. Excluding these left us with 180 clusters (see the “Data Availability” statement for online access to the clusters and stimulus materials). Finally, for each cluster we manually identified a key representative word, which was either the intuitive “name” of the group of words or a prototypical member. The resulting 180 target words consisted of 128 nouns, 22 verbs, 23 adjectives, 6 adverbs, and 1 function word. We selected five additional words from among the 20 most frequent cluster members, based on their prototypicality, to be used in generating the experimental materials.

To quantify the degree to which each dimension was spanned by the 180 key words, we defined a measure of dimension usage (Supplementary Figure 4). We consider each dimension “represented” by a word if the absolute value for that dimension in the vector for the word is in the top 10% of magnitude across the 29,805-word vocabulary. At least 5 words represented each dimension, and most dimensions had 10 or 20 words representing them (median = 16).

### Design of fMRI experiment 1 on words

For each of 180 target words, we created 6 sentences, 4–11 words long (mean = 6.85, st.dev. = 1.22), and each containing the target word used in the intended sense. Almost all sentences (1001/1080; 92.7%) contained at least one other representative word from the same cluster. We further found for each of 180 words, 6 images in the Google Image database. Thus, in the first two paradigms, each word appeared in a different sentence or with a different image across the repetitions of the word (Fig. 3). For the third paradigm, we selected five representative words from the cluster, and each word appeared with the same five words across repetitions, placed in a word cloud configuration around it. Please see the Data Availability statement for online access to the materials.

In the sentence paradigm, participants were asked to read the sentences and think about the meaning of the target word (in bold) in the context in which it is used. In the picture paradigm, participants were asked to read the word and think about its meaning in the context of the accompanying image. And in the third paradigm, participants were asked to read the target word (bolded, in the center of the word cloud) and to think about its meaning in the context of the accompanying words.

Within each scanning session, the 180 words were divided into two sets of 90 (done separately for each participant and paradigm) and distributed across two runs. Thus, it took two runs to get a single repetition of the full set of 180 words. Each participant saw between 4 and 6 repetitions for each of the three paradigms (Supplementary Table 5).

Across paradigms, each stimulus was presented for 3 s followed by a 2 s fixation period. Each run further included three 10 s fixation periods: at the beginning and end of the run, and after the first 45 trials. Each run thus took 8 min. Please see the Data Availability statement for online access to the presentation scripts.

### Design of fMRI experiments 2 and 3 on sentences

Experiment 2 used 96 passages, each consisting of 4 sentences about a particular concept, spanning a broad range of content areas from 24 broad topics (e.g., professions, clothing, birds, musical instruments, natural disasters, crimes, etc.), with 4 passages per topic (e.g., clarinet, accordion, piano, and violin for musical instruments; Supplementary Figure 1). All passages were Wikipedia-style texts that provided basic information about the relevant concept. Experiment 3 used 72 passages, each consisting of 3 or 4 sentences about a particular concept. As in experiment 2, the passages spanned a broad range of content areas from 24 broad topics, unrelated to the topics in experiment 2 (e.g., skiing, dreams, opera, bone fractures, etc.), with 3 passages per topic (Supplementary Figure 1). The materials included Wikipedia-style passages (*n* = 48) and first-/third-person narratives (*n* = 24). The two experiments were comparable in their within- and between-passage/topic semantic similarities (Fig. 5).

The sentences were 7–18 words long (mean = 11.8, st.dev. = 2.1) in experiment 2, and 5–20 words long (mean = 13.15, st.dev. = 2.92) in experiment 3. The passages in experiment 2 consisted of 4 sentences, and in experiment 3 they consisted of 3 or 4 sentences (mean = 3.375, st.dev. = 0.49). Sentences were presented in PsychToolbox font size 10 (variable width, average line on our display fits approximately 60 characters). If a sentence was longer than 50 characters, it was presented on 2 or, occasionally, 3 lines. The set of lines was always centered, horizontal and vertically.

In both experiments, participants were asked to attentively read the passages, presented one sentence at a time. The passages were divided into 8 sets of 12 (experiment 2) or 9 (experiment 3), corresponding to 8 runs. Thus, for each experiment, it took 8 runs to get a single repetition of the full set of 96/72 passages. Each participant did 3 repetitions (i.e., 24 runs, distributed across 3 scanning sessions). The division of passages into runs and the order was randomized for each participant and scanning session.

Each sentence was shown for 4 s followed by a 4 s fixation period. Each passage was further followed by another 4 s fixation period. Thus, each passage took 28 s (3-sentence passages) or 36 s (4-sentence passages). Each run further included a 10 s fixation at the beginning and end. The runs in experiment 2 were thus 452 s (7 min 32 s). Given that texts differed in length in experiment 3, and to make runs similar in duration, the texts were semi-randomly assigned to runs, so that the first 5 runs consisted of 6 three-sentence passages and 3 four-sentence passages for a total run duration of 296 s (4 min 56 s); and the last 3 runs consisted of 5 three-sentence passages and 4 four-sentence passages for a total run duration of 304 s (5 min 4 s).

Please see the Data Availability statement for online access to the materials and presentation scripts.

### Participants

Sixteen participants (mean age 27.7, range 21–50, 7 females), fluent speakers of English (15 native speakers, 1 bilingual with native-like fluency), participated for payment. Participants gave informed consent in accordance with the requirements of the Committee on the Use of Humans as Experimental Subjects (MIT) or Research and Integrity Assurance (Princeton). All 16 participants performed experiment 1 (three 2 h sessions), and the decoding results on their data were used to prioritize subjects for scanning on the other two experiments. Eight of the 16 participants performed experiment 2 (three 2 h sessions), and 6 of the 16 participants performed experiment 3 (three 2 h sessions). Four additional participants were scanned but not included in the analyses due to excessive motion and/or sleepiness during experiment 1 sessions. For two of these (scanned at Princeton), an incorrect echo-planar imaging (EPI) sequence was used, with no prospective motion correction; the other two were novice subjects (at MIT). These participants were excluded based on (i) visual inspection of framewise displacement^47^ over time to evaluate whether it exceeded 0.5 mm multiple times over the sentence or picture sessions (since at least those two were needed to train a decoder), and (ii) self-reports of sleepiness/difficulty paying attention.

### fMRI data acquisition and processing

Structural and functional data were collected on a whole-body 3-Tesla Siemens Trio scanner with a 32-channel head coil at the Athinoula A. Martinos Imaging Center at the McGovern Institute for Brain Research at MIT or at the Scully Center for the Neuroscience of Mind and Behavior at Princeton University. The same scanning protocol was used across MIT and Princeton. T1-weighted structural images were collected in 128 axial slices with 1 mm isotropic voxels (repetition time (TR) = 2530 ms, echo time (TE) = 3.48 ms). Functional, blood oxygenation level-dependent data were acquired using an EPI sequence (with a 90° flip angle and using GRAPPA with an acceleration factor of 2), with the following acquisition parameters: 31 4 mm thick near-axial slices, acquired in an interleaved order with a 10% distance factor; 2.1 mm × 2.1 mm in-plane resolution; field of view of 200 mm in the phase encoding anterior to posterior (A>P) direction; matrix size of 96 × 96 voxels; TR of 2000 ms; and TE of 30 ms. Further, prospective acquisition correction^51^ was used to adjust the positions of the gradients based on the participant’s motion one TR back. The first 10 s of each run were excluded to allow for steady-state magnetization.

MRI data were analyzed using FSL (http://fsl.fmrib.ox.ac.uk/fsl/)^48^ and custom MATLAB scripts. For each participant, we picked the structural scan in the sentence session as a reference and estimated a rigid registration of the structural scans from other sessions to it. The functional data from the runs in each scanning session were corrected for slice timing, motion, and bias field inhomogeneity and high-pass filtered (at 100 s cutoff). They were then registered to the structural scan in their own session, and thence to the reference structural scan (combining the two matrices), and finally resampled into 2 mm isotropic voxels. The reference structural scan was registered to the MNI template (affine registration+nonlinear warp), and the resulting transformation inverted to generate subject-specific versions of the various atlases and parcellations used. The responses to each stimulus were estimated using a general linear model (GLM) in which each stimulus presentation (sentence/word+picture/word+word cloud in experiment 1, and sentence in experiments 2–3) was modeled with a boxcar function convolved with the canonical haemodynamic response (HRF).

### Decoding methodology

The decoder operates by predicting a semantic vector given imaging data. Each dimension is predicted using a separate ridge regression with the regularization parameters estimated from training data. More formally, given a number of examples by number of voxels imaging data matrix *X* (training set), and the corresponding number of examples by number of dimensions semantic vector matrix *Z*, we learn regression coefficients ***b*** (a vector with as many entries as voxels) and *b*0 (a constant, expanded to a vector) that minimize

$$\left\| {{\boldsymbol{Xb}} + {\boldsymbol{b}}{\bf 0} - {\boldsymbol{z}}} \right\|_2^2 + \lambda \left\| {\boldsymbol{b}} \right\|_2^2$$

for each column ***z***—a semantic dimension—of the *Z* matrix.

The regularization parameter *λ* is set separately for each dimension using generalized cross-validation within the training set^49^. Each voxel was mean-normalized across training stimuli in each imaging experiment, as was each semantic vector dimension.

In experiment 1, the decoder was trained within a leave-10-words-out cross-validation procedure. In each fold, the regression parameters for each dimension were learned from the brain images for 170 words, and predicted semantic vectors generated from the brain images for 10 left-out words. The voxelwise normalization was carried out using a mean image derived from the training set, which was also subtracted from the test set. The cross-validation procedure was carried out on data of each of the three paradigms separately or on the dataset resulting from averaging them; we report results for all of these. This resulted in 180 decoded semantic vectors. In experiments 2 and 3, the decoder was trained on brain images for 180 words from experiment 1 and applied to brain images of 384 and 243 sentences, respectively, resulting in 384 and 243 decoded semantic vectors.

Each decoder was trained on images reduced to a subset of 5000 voxels, approximately 10% of the number left after applying a cortical mask. We picked this number as a conservative upper bound for the number of informative voxels, as determined in previous studies^20^. Voxels were selected by the degree to which they were informative about the text-derived semantic vectors for training set images, as measured by ridge regressions on the voxel and its adjacent three-dimensional (3D) neighbors. We learned ridge regression models (regularization parameter set to 1) to predict each semantic dimension from the imaging data of each voxel and its 26 adjacent neighbors in 3D, in cross-validation within the training set. This yielded predicted values for each semantic dimension, which were then correlated with the values in the true semantic vectors. The informativeness score for each voxel was the maximum such correlation across dimensions.

In experiment 1, voxel selection was done separately for each of the 18 cross-validation folds, with a nested cross-validation inside each 170 concept training set (any two training sets share ~95% of concepts). In experiments 2 and 3, voxel selection was done using the entire 180 concept dataset used to train the decoder.

### Statistical testing of decoding results

To obtain the decoding results reported in Fig. 4a, we carried out pairwise classification on decoded vectors and the corresponding text-derived semantic vectors. The set of pairs considered varies across experiments and classification tasks. In each pair, we calculated the correlation between the two decoded vectors and the two text-derived semantic vectors. Classification was deemed correct if the highest correlation was between a decoded vector and the corresponding text semantic vector. The accuracy values reported are the fractions of correctly classified pairs. For experiment 1, we compared every possible pair out of 180 words. For experiments 2 and 3, we compared every possible pair of sentences where sentences came from (i) different topics, (ii) different passages within the same topic, and (iii) the same passage.

All accuracy values were tested with a binomial test^38^, which calculates:

$$P(X \ge {\mathrm{number}}\,{\mathrm{of}}\,{\mathrm{correct}}\,{\mathrm{pairs}}\left| {H_0} \right.:{\mathrm{classifier}}\,{\mathrm{at}}\,{\mathrm{chance}}\,{\mathrm{level}})$$

and requires specification of the number of independent pairs being tested. As results are correlated across all pairs that involve the same concept or sentence (they all share the same decoded vector), we used extremely conservative values. For experiment 1, we used 180, the number of distinct words. For experiments 2 and 3, we used (i) the number of passage pairs across different topics, multiplied by the minimum number of sentences per passage, (ii) the number of passage pairs within the same topic, multiplied by number of topics and the minimum number of sentences per passage, and (iii) the number of sentences.

The rank accuracy results reported in Fig. 4b were obtained by comparing each decoded vector to a decoding range, a set of candidate vectors corresponding to all the possible stimuli in experiments 1 (180 words), 2 (384 sentences), and 3 (243 sentences). We then calculate the rank of the correct stimulus in that range, and average it across all decoded vectors. This average rank score is normalized into a rank accuracy score, $1 - \frac{{\left\langle {\mathrm{rank}} \right\rangle - 1}}{{\left\langle {\# {\mathrm{vectors}}\,{\mathrm{in}}\,{\mathrm{range}}} \right\rangle - 1}}$.

The rank accuracy score is in [0,1], with 1 corresponding to being at the top of the ranking and 0 at the bottom; chance level performance is 0.5. The rank accuracy score is commonly used in information retrieval in situations where there are several elements in a range that are similar to the correct one. It becomes the usual accuracy if there are two elements in the range. The null model for chance level performance treats each ranking as a multinomial outcome, which is then normalized to a rank accuracy score. Using the Central Limit Theorem, the average of the scores has a normal distribution, with mean 0.5 and variance $\frac{{\# {\mathrm{vectors}}\,{\mathrm{in}}\,{\mathrm{range}} + 1}}{{12\left( {\# {\mathrm{vectors}}\,{\mathrm{in}}\,{\mathrm{range}} - 1} \right)\# tests}}$. Again, as the outcomes for different sentences in the same passage could possibly be correlated, we used a conservative value for the number of tests (number of passages rather than number of sentences).

The word-spotting results were obtained by comparing each decoded vector to the vectors for all words in a basic vocabulary of ~30 K words, calculating the rank accuracy of each word in the corresponding stimulus sentence, and taking the maximum of those as the word-spotting score. Given that, for different subjects, the sentences with high scores might be different (as might the words within each sentence), we opted to compare the distribution of scores in each subject against the distribution under a null model. We obtain the null model in this case by simulating as many multinomial draws as there are words in each sentence, normalizing them, and calculating the word-spotting score for the sentence by taking the highest value. In each simulation run we obtain a histogram of results, and we average these bin-wise across 1000 runs to yield a distribution under the null model (under the Central Limit Theorem that suffices to get a precise estimate of bin counts). This is compared with the average of the subject histograms in Fig. 4c. To produce *p*-values under the null hypothesis we tested the histogram of each subject against the null model histogram, using a two-sided Kolmogorov–Smirnov test. Finally, in all three measures we applied a Bonferroni multiple comparison correction, accounting for the number of subjects and classification tasks.

For the comparison between results obtained selecting voxels from anywhere in the brain and selecting them while restricting them to different networks, we used two separate tests. For pairwise accuracy, we used a one-sided paired *t*-test, with each sample containing the average accuracy for pairs involving each sentence, for all subjects (sample sizes were 3072 and 1458 for experiments 2 and 3, respectively). For the rank accuracy results we used a simple sign test with samples containing the rank accuracy results for all subjects. While testing for significance, we applied Bonferroni correction taking into account the number of classification tasks (3 pairwise accuracy+1 rank accuracy), networks (5), and experiments (2).

### Model building approaches

In order to provide a more structured perspective of the commonalities and differences between related studies and ours, we highlight the key study characteristics in Supplementary Tables 6 and 7

Supplementary Table 6 contrasts the studies in terms of (i) the type of model being learned, (ii) what is being predicted, and (iii) the task used to quantitatively evaluate the prediction. In all studies, the model building (training) stage consists of learning a relationship between the representations of input stimuli and the imaging data. This relationship is then used to make a prediction about new stimuli during the test stage. This is, typically, either a prediction of (i) the imaging data (on a voxel-by-voxel basis) in response to test stimuli, or (ii) the representation of the stimulus presented when test imaging data were acquired. Both types of prediction lend themselves naturally to pairwise classification as an evaluation task, especially in cross-validation approaches, as it is straightforward to leave out two test stimuli (and corresponding imaging data) and build a model from the remainder. This task is also appealing because the statistical testing of its results is well understood, given enough precautions to ensure the independence of training and test data^50^. The studies that, like ours, extract a representation of the stimulus allow for a wider range of evaluation strategies beyond the pairwise classification approach, including, for example, comparing the semantic vector extracted to those of hundreds of candidate concepts and sentences, using rank accuracy, or generating an approximate reconstruction of the stimulus. For ease of comparison across studies, we focus on classification tasks in Supplementary Table 6.

Supplementary Table 7 contrasts the studies in terms of the type and range of stimuli used. For evaluating the ability of the decoder to generalize to new stimuli, the training and testing data ideally come from separate experiments, and even different experimental paradigms. If this is not feasible, then the most common approach is cross-validation, where a portion of the data is used as training data, and the remainder of the data (from the same experiment) as test data. The latter is the strategy used in all the studies that have included a single experiment. A handful of studies have included two experiments (one for training and one for testing). In contrast, our study consisted of five experiments (three used in combination for training the model and two separate test experiments). For ease of comparison with prior work, we included a cross-validated evaluation within our single-word experiment (experiment 1), in addition to reporting the results for the two separate test experiments (experiments 2 and 3). To our knowledge, our study is the first to show generalization from individual words to sentence stimuli across scanning sessions.

### Code availability

The stimulus presentation code is available via the paper website (https://osf.io/crwz7). The only custom code used to produce results was (i) code for training the decoder from imaging data (implementing a well-known regression approach^51^) and (ii) code for identifying informative voxels (with a similar regression model applied to voxels and their spatial neighbors). Both of these functions are available in the paper website.

### Data availability

The imaging datasets used for experiments 1, 2, and 3 are publicly available on the paper website (https://osf.io/crwz7). Given the size of the datasets (~150 2 h imaging sessions), we provide MATLAB data containing solely deconvolved images and labels for each stimulus (concept or sentence). The raw and processed NIFTI imaging datasets, as well as associated event files, will be shared via a repository (http://www.openfmri.org), after re-processing. Any updates on the data and scripts will be posted on the paper website (https://osf.io/crwz7).

## Electronic supplementary material

Supplementary Information
