# Supplementary material for: Toward a universal decoder of linguistic meaning from brain activation
Source: Nat Commun. 2018 Mar 6;9:963. doi: 10.1038/s41467-018-03068-4 (PMC5840373; doi:10.1038/s41467-018-03068-4)
Supplement: Supplementary file 1 — Supplementary Information [file 41467_2018_3068_MOESM1_ESM.pdf]

Supplementary Information

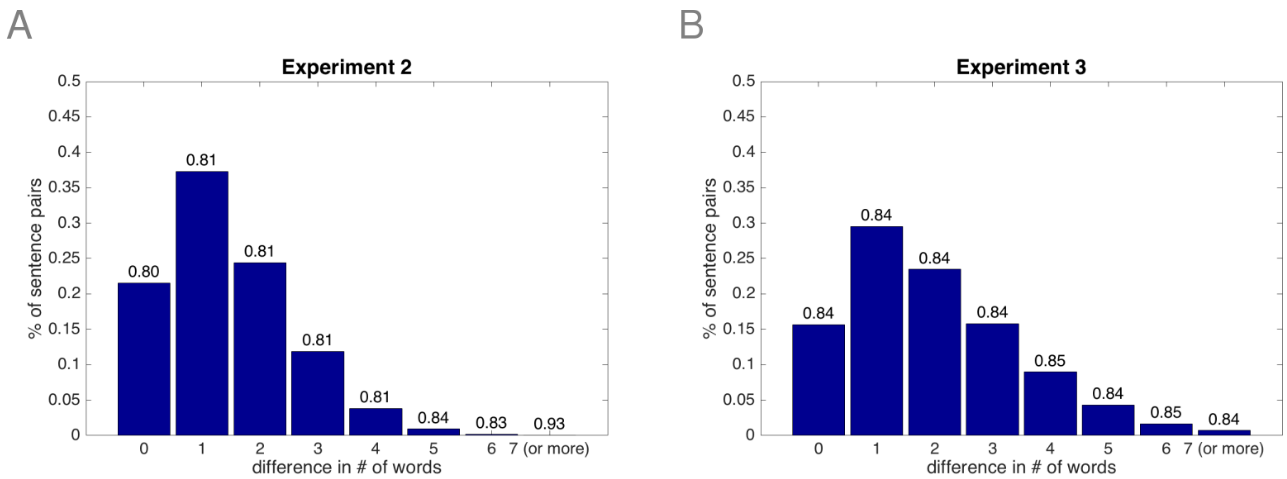

Supplementary Figure 1. A) Distribution of the difference in the number of content words across pairs of sentences in Experiment 2 (~20% of pairs have the same number of words, ~36% differ by one word, etc). The plot also shows the average accuracy across sentence pairs with a given difference, above the corresponding bar; this is also averaged across subjects. B) Same plot for Experiment 3.

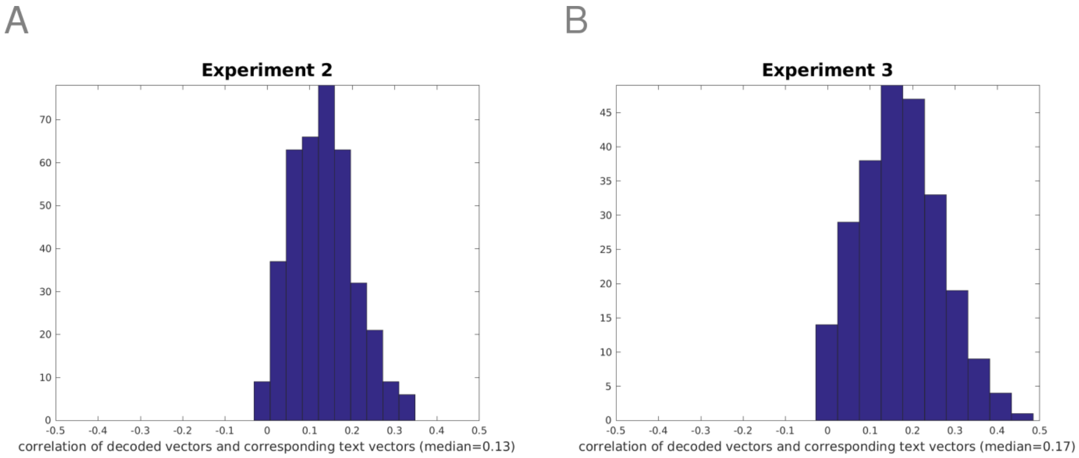

Supplementary Figure 2. A) Distribution of correlation between decoded and text vectors for the matching stimuli in Experiment 2 (the diagonal of Figure 5). B) Same plot for Experiment 3.

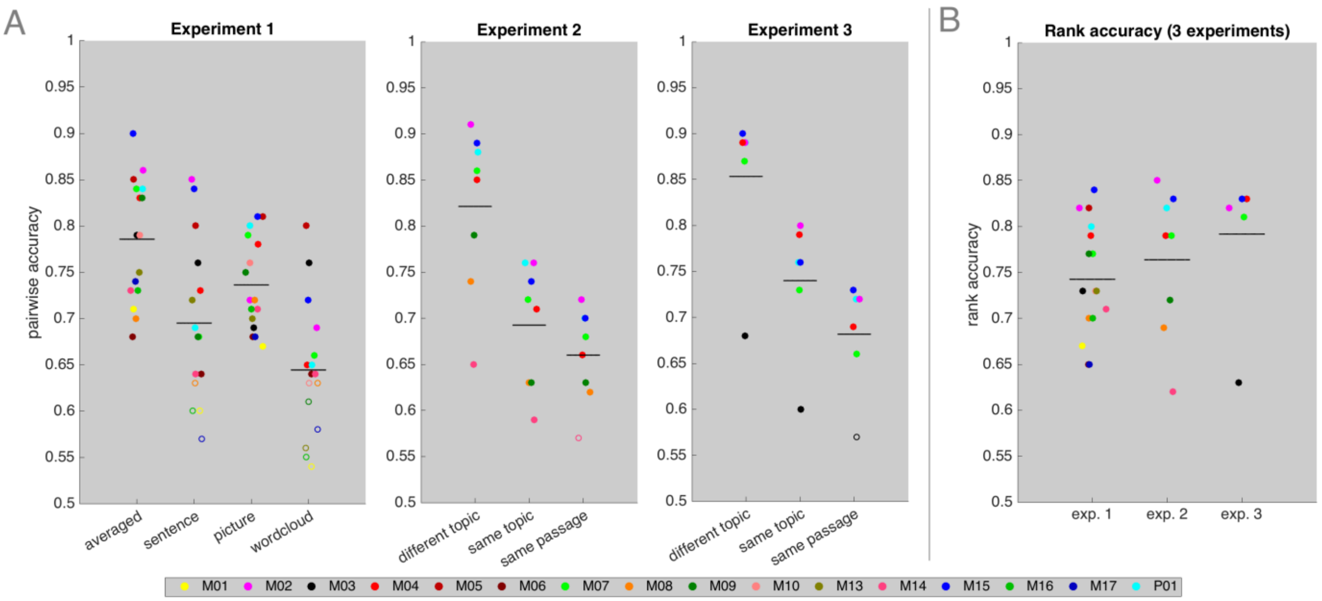

Supplementary Figure 3. Results obtained using Skip-Thought vectors to represent word and sentence meanings (cf. averaging the content word vectors, as in Figure 4). **A)** Pairwise accuracy results in Experiments 1 (n=16, left), 2 (n=8, middle), and 3 (n=6, right). For Experiment 1, we report results using each of the three paradigms, as well as the average of the three paradigms. For Experiments 2 and 3, we report three measures: classifying sentences from i) different topics (left), ii) different passages within the same topic (middle), and iii) the same passage (right). Each dot represents pairwise accuracy for an individual subject. Dots of the same color refer to the same individual across experiments. Filled dots (cf. empty dots) represent significant results. B) Rank accuracy results in Experiments 1 (180 word choices), 2 (384 sentence choices), and 3 (243 sentence choices).

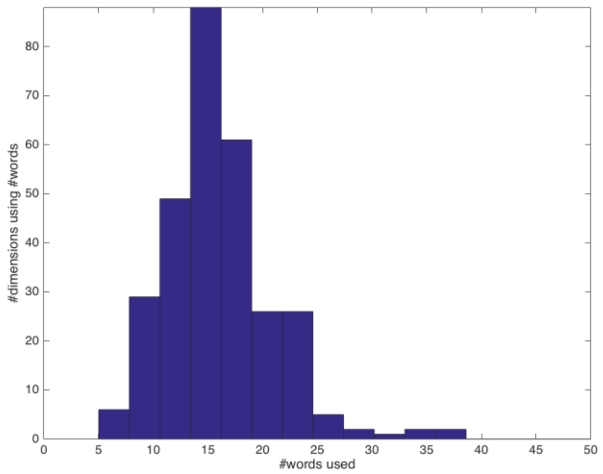

Supplementary Figure 4. The distribution of the number of words (out of the 180) that use each dimension of the semantic space (minimum 5, median 16, maximum 38).

	pairwise classification				rank classification
participants	average	sentence	picture	wordcloud	180 concepts
M01	2.18E-07		1.81E-05		2.00E-15
M02	1.00E-16	1.00E-16	2.77E-09	1.05E-06	1.00E-16
M03	6.05E-12	6.05E-12	1.05E-06	6.05E-12	1.00E-16
M04	1.00E-16	5.21E-11	1.97E-12	3.48E-05	1.00E-16
M05	1.00E-16	1.11E-16	1.11E-16	1.11E-16	1.00E-16
M06	2.18E-07		2.18E-07		2.06E-12
M07	1.22E-15	1.05E-06	1.11E-16	1.20E-04	1.00E-16
M08	2.77E-09		2.77E-09		1.00E-16
M09	1.11E-16	1.05E-06	5.21E-11		1.00E-16
M10	1.97E-12	2.18E-07	6.05E-12		1.00E-16
M13	1.97E-12	2.77E-09	2.18E-07		1.00E-16
M14	6.05E-12		9.59E-08		1.00E-16
M15	1.00E-16	1.00E-16	1.11E-16	1.72E-08	1.00E-16
M16	4.01E-10		9.59E-08		1.00E-16
M17	1.81E-05		1.05E-06		7.07E-14
P01	1.00E-16	2.18E-07	1.65E-14	3.48E-05	1.00E-16

Supplementary Table 1. Experiment 1) p-values for all the significant results on pairwise and rank classification tasks reported in the main text, for all participants. A p-value is omitted if the result is not significant (<0.01 after Bonferroni correction for the number of participants and tasks). A p-value is listed as 1E-16 if smaller than precision can represent.

	pairwise classification			rank classification
participants	different topic	same topic	same passage	384 sentences
P01	1.00E-16	1.00E-16	1.00E-16	1.00E-16
M02	1.00E-16	1.00E-16	1.00E-16	1.00E-16
M04	1.00E-16	1.00E-16	2.39E-10	1.00E-16
M07	1.00E-16	1.00E-16	7.89E-13	1.00E-16
M08	1.00E-16	9.02E-08	1.06E-05	5.52E-10
M09	1.00E-16	2.22E-16	2.39E-10	6.41E-12
M14	1.00E-16	1.18E-14	1.06E-05	1.91E-07
M15	1.00E-16	1.00E-16	7.89E-13	1.00E-16

Supplementary Table 2. Experiment 2) p-values for all the significant results on pairwise and rank classification tasks reported in the main text, for all participants. A p-value is omitted if the result is not significant (<0.01 after Bonferroni correction for the number of participants and tasks). A p-value is listed as 1E-16 if smaller than precision can represent.

	pairwise classification			rank classification
participants	different topic	same topic	same passage	243 sentences
P01	1.00E-16	1.00E-16	1.00E-16	1.00E-16
M02	1.00E-16	1.00E-16	2.22E-16	1.00E-16

M03	1.00E-16	2.05E-05	1.55E-04	2.08E-05
M04	1.00E-16	1.00E-16	4.44E-07	1.00E-16
M07	1.00E-16	1.00E-16	1.12E-07	1.11E-16
M15	1.00E-16	1.00E-16	4.44E-14	1.00E-16

Supplementary Table 3. Experiment 3) p-values for all the significant results on pairwise and rank classification tasks reported in the main text, for all participants. A p-value is omitted if the result is not significant (<0.01 after Bonferroni correction for the number of participants and tasks). A p-value is listed as 1E-16 if smaller than precision can represent.

		brain	language	default	task	visual	other
	approximate # voxels	~50K	4670	6490	11630	8170	~20K
	(average across 16 subjects)			(Power 2011, minus any language overlap)			
Experiment 2	pairwise: different topic		6.25E-08	2.22E-26	2.40E-14	5.22E-39	4.42E-22
	pairwise: different passage		4.18E-04	2.00E-22	3.68E-06	1.13E-17	4.87E-18
	pairwise: same passage		5.63E-05	7.14E-18	6.30E-10	1.49E-33	1.30E-15
	rank: 384 sentences		3.91E-03	3.91E-03	3.91E-03	3.91E-03	3.91E-03
Experiment 3	pairwise: different topic		2.69E-15	1.81E-26	2.21E-18	1.08E-38	7.13E-19
	pairwise: different passage		1.33E-03	1.13E-09	1.04E-07	1.02E-11	1.28E-07
	pairwise: same passage		3.80E-01	9.23E-08	3.14E-04	2.43E-09	1.47E-04
	rank: 243 sentences		1.56E-02	1.56E-02	1.56E-02	1.56E-02	1.56E-02

Supplementary Table 4: Top row: Approximate numbers of voxels for the whole brain, language network, the three Power 2011 networks described in the text, and the rest of the brain after excluding those four networks. Bottom rows: p-values comparing decoding performance selecting 5000 voxels from anywhere in the brain, to that obtained restricting the choice of voxels to each of the four networks, or the rest of the brain, as reported in Table 1. The p-values < 0.01 after Bonferroni correction are shown in bold.

SUB_ID	Sentences	Images	Word clouds
M01	5	5	4
M02	5	5	5
M03	5	5	5
M04	4	6	5
M05	4	5	4
M06	5	6	5
M07	5	6	5
M08	6	5	5

M09	5	6	6
M10	6	4	5
M13	5	6	5
M14	6	5	5
M15	6	5	6
M16	5	5	5
M17	4	6	5
P01	5	4	5

Supplementary Table 5: Number of repetitions per word per paradigm in each participant in Experiment 1.

Supplementary Table 6. A summary of prior related literature, describing the architecture and predictions made by each decoding system, and its quantitative evaluation task and results. Reference numbers - except Supplementary Reference 52 - refer to references in the main text.

Citation	Model being learned	Prediction from model	Quantitative decoding task (s)	Average score(s) across subjects
Thirion et al.[11]	Model each voxel as a linear function of filter outputs (learn function coefficients and filter parameters)	Predict the pattern of activation for each test stimulus from the decoding experiment	6-Way classification of test stimulus by comparing the predicted pattern of activation against the true patterns	83%
Kay et al.[12]	Model each voxel as a linear function of filter outputs (learn function coefficients and filter parameters)	Predict the pattern of activation for each test stimulus from the decoding experiment	120-Way classification of test stimulus by comparing the predicted pattern of activation against the true patterns (potentially many more)	42% (single trial) and 82% (average of 13 trials) accuracy
Miyawaki et al.[13]	Model each voxel as a linear function of filter outputs (learn function coefficients)	Predict the pattern of activation for each test stimulus from the decoding experiment	100-Way classification of test stimulus by comparing the predicted pattern of activation against the true patterns (potentially many more)	90% (single volume) and 98% (block average accuracy), 0.66 correlation between predicted and true
Mitchell et al.[16]	Model each voxel as a linear function of the semantic vector (learn function coefficients)	Predict the pattern of activation for two left-out stimuli	2-Way classification of left-out stimuli by comparing the predicted pattern of activation against the true patterns	72%
Naselaris et al.[14]	Probabilistic model of each voxel using either structural or semantic representations	Posterior probability model over stimulus image given test pattern of activation (and using flat, gabor, or natural image database as priors)	23-Way classification (probability that a reconstruction obtained using some specific reconstruction method would belong to the same semantic category as the target image)	40%
Palatucci et al.[17]	Model semantic vectors as linear functions of the pattern of activation across a subset of voxels	Predict the semantic vectors for two left-out stimuli	2-Way choice using a nearest neighbor classifier with the semantic vectors for two left-out stimuli (and some results on larger concept ranges)	70% (5000-D) and 81% (218-D)
Just et al.[18]	Model each voxel as a linear function of the semantic vector (learn function coefficients)	Predict the pattern of activation for two left-out stimuli	2-Way classification of left-out stimuli by comparing the predicted pattern of activation against the true patterns	80%
Nishimoto et al.[15]	Model each voxel as a linear combination of filter outputs (passed through an hemodynamic response filter)	Predict the pattern of activation for a given test stimulus snapshot	(1) Correlation between predicted and observed activation patterns in early visual areas over test stimuli, and (2) classification of patterns of activation for 1T test stimuli into 3 TR bins (out of 162 possible), by comparing the predicted pattern of activation against the true patterns (potentially many more)	(1) Correlation: 0.24, 0.39, and 0.40 (static, nondirectional, and directional encoding models), (2) accuracy: 95%
Pereira et al.[19,20]	Model semantic vectors as linear functions of the pattern of activation across a subset of voxels	Predict the semantic vectors for two left-out stimuli	2-Way choice using a nearest neighbor classifier with the predicted semantic vectors for left-out stimuli (other results on larger concept ranges and between-category predictions)	79% (75-D)
Huth et al.[52]	Model each voxel as a linear function of the semantic features (learn function coefficients)	Predict the pattern of activation for a given sequence of semantic feature vectors for the test stimuli	52-Way identification of segment of test data by comparing predicted pattern of activation over 9TRs against true patterns	76%

Supplementary Table 6 (continued)

Citation	Model being learned	Prediction from model	Quantitative decoding task (s)	Average score(s) across subjects
Wehbe et al.[23]	Model each voxel at a given time point as a linear combination of semantic features during the preceding four time points	Predict the pattern of activation for left-out text passages (4 TRs, 16 words)	2-Way classification of left-out stimuli by comparing the predicted pattern of activation across time against the true patterns	74%
Handjaras et al.[21]	Model each voxel as a linear function of the semantic vector (learn function coefficients)	Predict the pattern of activation for two left-out stimuli	2-Way classification of left-out stimuli by comparing the predicted pattern of activation against the true patterns	77% (pictures), 63% (words), 66% (read aloud)
Huth et al.[24]	Model each voxel at a given time point as a linear combination of semantic features during the preceding four time points	Prediction of patterns of activation during test story, given its text	correlation between predicted and actual response for each voxel	(Correlation between predicted and actual significant in many brain areas)
Anderson et al.[25]	Model each voxel as a linear function of the semantic vector (learn function coefficients); the imaging data used are word activation patterns obtained by averaging activation patterns for all sentences containing the word	Predict the pattern of activation for two left-out stimuli given semantic vector	2-Way classification of left-out sentences by comparing the predicted pattern of activation against the true patterns	62% (whole cortex) or 72% (LSTS)
Anderson et al.[22]	Calculate similarity matrices between (1) all activation patterns for words, and (2) all semantic/visual vectors for words (and possibly average resulting matrices)	For two left-out stimuli, calculate their similarity profile to all other 68 from the activation data	2-Way classification task representational similarity space (match the similarity profiles derived from activation to those derived from semantic or visual vectors)	67% (visual), 76% (text), 77% (both)
Wang et al.[26]	Model each voxel as a linear function of the semantic vector (learn function coefficients); the imaging data used are word activation patterns obtained by averaging activation patterns for all sentences containing the word	Predict the pattern of activation for each of 240 sentences, given their semantic vectors	240-Way classification task (compare the pattern of activation for a left-out sentence to the true patterns for 240 sentences); rank accuracy measure	82%
This paper	Model semantic vectors as linear functions of the pattern of activation across a subset of voxels	Predict the semantic vectors for two left-out stimuli	2-Way choice using a nearest neighbor classifier with the predicted semantic vectors for left-out stimuli	79% (concept decoding)<br>83% (sentences with different topics)<br>73% (sentences in passages with the same topic)<br>69% (sentences in the same passage)
		Predict the semantic vector for a left-out stimulus	180-Way, 384-way, and 243-way choices using a nearest neighbor classifier with the predicted semantic vectors for left-out stimulus; rank accuracy measure	77% (concept decoding)<br>76% (384 sentences)<br>79% (243 sentences)

Supplementary Table 7. A summary of prior related literature, describing both the training/test stimuli used and the way that they were represented inside the decoding system described in each paper. Reference numbers - except Supplementary Reference 52 - refer to references in the main text.

Citation	Stimuli	Stimulus details	Representation of stimuli	No. of experiments	No. of subj.
Thirion et al.[11]	Binary patterns	Decoding: 3×3 grid binary contrast patterns, model: conventional retinotopy patterns	Output of visual field filter bank	2 (model building and decoding)	8
Kay et al.[12]	Natural images	Model: 1750 images, decoding: 120 images	Gabor filter pyramid (sensitive to location, orientation and spatial frequency)	2 (model building and decoding)	3
Miyawaki et al.[13]	Binary figures	Decoding: 10×10 grid random/figure binary contrast patterns model: conventional retinotopy patterns	Output of visual field (multiscale) filter bank	2 (model building and decoding)	2
Mitchell et al.[16]	Words + line drawing of concept named by each word	60 Concrete nouns: 5 exemplars from 12 semantic categories (vegetables, animals, insects, body parts, tools, clothing, objects (kitchen), objects (other), furniture, buildings, building parts, vehicles)	25-Dimensional semantic vector (derived from text corpus co-occurrence counts of concept name with 25 hand-picked verbs)	See Mitchell et al.[16]	9
Naselaris et al.[14]	See Kay et al.[12]	See Kay et al.[12]	Structural (see Kay et al.[12]) and semantic (23 possible semantic category labels)	See Kay et al.[12]	3
Palatucci et al.[17]	See Mitchell et al.[16]	See Mitchell et al.[16]	5000-Dimensional semantic vector (derived from text corpus co-occurrence counts of concept name with 5000 most frequent words), 218-dimensional semantic feature vector (derived from AMT subjects)	See Mitchell et al.[16]	9
Just et al.[18]	Words	See Mitchell et al.,[16]	3-Factor semantic vector (shelter, manipulation, eating) + word length	1 (decode on 2 left-out stimuli, build model from the rest)	11
Nishimoto et al.[15]	Movie clips	Model: 120 min of 10-20 s clips, shown once, decoding: 9 1-min movies, shown 10×	Bank of motion energy filters (nonlinear, stages uses gabor filter pyramids)	2 (model building and decoding)	3
Pereira et al.[19,20]	See Mitchell et al.[16]	See Mitchell et al.[16]	50- Or 75-dimensional semantic vectors (derived from the Wikipedia page with the same title)	See Mitchell et al.[16]	9
Huth et al.[52]	Movie clips	Model: see Nishimoto et al.[15], decoding: 10 1-min movies, shown 10×	1705-Dimensional binary semantic feature vector (derived from presence of 1364 common objects (nouns) and actions (verbs) from the WordNet lexicon + 341 higher-order categories inferred via WordNet) + feature for total motion energy during each second of the movie	2 (model building and decoding)	5
Wehbe et al.[23]	Text passages comprising a chapter of Harry Potter and the Sorcerer's Stone (presented in 0.5 s RSVP)	5000 Words	195-Dimensional feature vector for each word (including: semantic vectors derived from word dependency and co-occurrence counts, number of letters in the individual word, part of speech and role in the parse of the sentence it appears in, encoding of the emotions	1 (decode on 2 left-out stimuli, build model from the rest)	8

Supplementary Table 7 (continued)

Citation	Stimuli	Stimulus details	Representation of stimuli	No. of experiments	No. of subj.
			and events involving different story characters)		
Handjaras et al.[21]	Words (concrete, presented as visual picture, visual word, or auditory word)	40 Concepts from 8 categories (vegetables, fruits, natural and artificial places, mammals, birds, tools, vehicles)	812/743-Dimensional binary feature vector for each concept (features are properties, elicited from sighted or blind subjects, respectively)	3 (model built and tested within each experiment)	26 sighted, 22 blind
Huth et al.[24]	Stories (presented auditorily)	Model: 10 10–15 min stories, shown once, decoding: one 10 min story, shown 2×	1026-Dimensional feature vector for each word (including: 985-dimensional semantic vectors derived from text corpus co-occurrence counts of stimulus words and 985 most frequent words; 39 dimensions for word frequency, phoneme rate, phonemes present)	2 (model building and decoding)	7
Anderson et al.[25]	Sentences	240 Sentences combining 141 nouns, 62 verbs, and 39 adjectives (0.4 s RSVP word presentation); semantic relatedness word probe after some sentences	65-Dimensional semantic vector for each concept (real-valued dimensions reflect sensory, motor, affective and other types of information encoded in the brain; values elicited by showing the word naming a concept in AMT); sentences are represented by the average vector of their content words	1 (decode on 2 left-out stimuli, build model from the rest)	14
Anderson et al.[22]	Words	70 Words in 7 categories (attribute, communication, event/action, person/social-role, location, object/tool, ur-abstract) from 2 domains (law and music)	300-Dimensional semantic vector (word2vec skipgram with negative sampling, trained on English and Italian wikipedias), visual vector (pre-softmax layer from a CNN trained on Imagenet, operating on 20 Google Images results per word)	1 (decode on 2 left-out stimuli, build model from the rest)	9
Wang et al.[26]	Sentences	240 Sentences combining 141 nouns, 62 verbs, and 39 adjectives (sentences presented for 5 s)	42-Dimensional semantic vector for each concept (binary dimensions reflect the presence/absence of perceptual and affective characteristics, as well as functional, temporal, spatial, and other properties; values elicited by showing the word naming each concept to three raters); sentences are represented by the average vector of their content words	1 (decode on left-out stimulus, build model from the rest)	7
This paper	Sentences, words+pictures, word clouds	180 Words, concreate and abstract (6 sentences about each, word paired with picture, word and cloud of related words)	300-Dimensional semantic vector (Global Vector, trained on 42B token corpus)	3 (model building)	16
	Sentences	384 Sentences (in 96 passages)	Sentences are represented by the average vector of their content words	1 (decoding)	8
		243 Sentences (in 72 passages)		1 (decoding)	6

Supplementary References:

52. Huth, A. G., Nishimoto, S., Vu, A. T. & Gallant, J. L. A continuous semantic space describes the representation of thousands of object and action categories across the human brain. *Neuron* **76**, 1210-1224 (2012).
